# Up-Regulation of hERG K^+^ Channels by B-RAF

**DOI:** 10.1371/journal.pone.0087457

**Published:** 2014-01-27

**Authors:** Tatsiana Pakladok, Zohreh Hosseinzadeh, Ahmad Almilaji, Aleksandra Lebedeva, Ekaterina Shumilina, Ioana Alesutan, Florian Lang

**Affiliations:** 1 Department of Physiology, University of Tübingen, Tübingen, Germany; 2 Department of Immunology, Institute of Experimental Medicine, St. Petersburg, Russia; Dalhousie University, Canada

## Abstract

Human ether-a-go-go related-gene K^+^ channels (hERG) participate in the regulation of tumor cell proliferation and apoptosis. HERG channel activity is up-regulated by growth factors. Kinases sensitive to growth factor signaling include the serine/threonine protein kinase B-RAF. The present study thus explored whether B-RAF influences hERG channel expression and activity. To this end, hERG channels were expressed in *Xenopus* oocytes with or without wild-type B-RAF, hERG channel activity was determined utilizing dual-electrode voltage clamp and hERG protein abundance in the cell membrane was analyzed utilizing confocal microscopy as well as chemiluminescence. Moreover, in rhabdomyosarcoma RD cells the effect of B-RAF inhibitor PLX-4720 on hERG-mediated current was quantified by whole-cell patch clamp and hERG cell surface protein abundance by utilizing biotinylation of cell surface proteins as well as flow cytometry. As a result, co-expression of wild-type B-RAF in hERG-expressing *Xenopus* oocytes significantly increased hERG channel activity and hERG channel protein abundance in the cell membrane. Treatment for 24 hours of B-RAF and hERG-expressing *Xenopus* oocytes with B-RAF inhibitor PLX-4720 (10 µM) significantly decreased hERG-mediated current and hERG cell surface expression. Similarly, in rhabdomyosarcoma RD cells, treatment for 24 hours with B-RAF inhibitor PLX-4720 significantly decreased hERG cell membrane protein abundance and hERG-mediated current. In conclusion, B-RAF is a powerful regulator of hERG channel activity and cell surface hERG protein abundance.

## Introduction

Human ether-a-go-go related-gene K^+^ channels, hERG (encoded by the KCNH2 gene) are expressed strongly in the heart and contribute to the repolarization of cardiomyocytes [1]–[4]. Beyond that, the hERG K^+^ channels are expressed in a variety of tumor cells and participate in the machinery regulating tumor cell proliferation [5]–[9], apoptosis [10], [11] as well as tumor cell migration [12], [13]. Accordingly, inhibitors of hERG channels have been considered for the treatment of malignancy [5], [6], [9], [14]–[16].

HERG K^+^ channels are up-regulated by and contribute to the signaling of growth factors [17]–[19]. Growth factor signaling involves also B-RAF [20], a serine/threonine kinase up-regulated in a variety of tumor cells [21]–[24]. The kinase plays a critical role in the activation of the RAS/RAF/MEK/ERK pathway, which participates in the regulation of cell proliferation, differentiation and survival [25].

In view of the importance of hERG channels in tumor cell proliferation and apoptosis and considering that B-RAF is up-regulated in tumor cells, we hypothesized that B-RAF may participate in the regulation of these channels. In order to test this hypothesis, hERG was expressed in *Xenopus* oocytes with or without additional co-expression of wild-type B-RAF. hERG channel activity was determined utilizing dual-electrode voltage clamp and hERG channel protein abundance in the cell membrane by chemiluminescence and immunocytochemistry with subsequent confocal microscopy. HERG channel activity in hERG and B-RAF-expressing *Xenopus* oocytes was further determined following treatment with B-RAF inhibitor PLX-4720 which has been considered as a potent anti-proliferative and pro-apoptotic drug for the treatment of cancer [26]. Moreover, biotinylation of cell surface proteins and flow cytometry as well as whole-cell patch clamp experiments have been employed to quantify the effect of PLX-4720 on hERG cell membrane expression and hERG mediated current in rhabdomyosarcoma RD cells. As a result, B-RAF is indeed a powerful regulator of hERG K^+^ channels.

## Materials and Methods

All animal experiments were conducted according to the recommendations of the Guide for Care and Use of Laboratory Animals of the National Institutes of Health as well as the German law for the welfare of animals, and reviewed and approved by the respective government authority of the state Baden-Württemberg (Regierungspräsidium) prior to the start of the study (Anzeige für Organentnahme nach §6). The *Xenopus* oocytes were explanted from adult *Xenopus laevis* (NASCO, Fort Atkinson, USA). The frogs were anaesthesized by a 0.1% Tricain solution. After confirmation of anaesthesia and disinfection of the skin, a small abdominal incision was made and oocytes were removed, followed by closure of the skin by sutures. All efforts were made to minimize animal suffering.

For generation of cRNA, constructs encoding hERG [27], hERG-HA containing an extracellular hemagglutinin epitope [28], and human wild-type B-RAF [29] were used. cRNA synthesis, handling and injection of *Xenopus* oocytes have been described previously in detail [30]–[32]. Where indicated, *Xenopus* oocytes were first injected with water or 10 ng cRNA encoding wild-type B-RAF and then with 10 ng cRNA encoding hERG, later on the same day. Standard two-electrode voltage clamp recordings were performed three days after cRNA injection [33], [34]. The oocytes were maintained at 17°C in a ND96 solution containing: 88.5 mM NaCl, 2 mM KCl, 1 mM MgC1_2_, 1.8 mM CaC1_2_, 5 mM HEPES, 0.11 mM tetracycline (Sigma, Steinheim, Germany), 4 µM ciprofloxacin (Fresenius, Bad Homburg, Germany), 0.2 mM refobacin (MerckSerono, Darmstadt, Germany), 0.5 mM theophylline (Takeda, Singen, Germany) and 5 mM sodium pyruvate (Sigma, Steinheim, Germany), pH 7.4 adjusted with NaOH [35]. The control solution contained: 96 mM NaCl, 2 mM KCl, 1.8 mM CaCl_2_,1 mM MgCl_2_ and 5 mM HEPES, pH 7.4 adjusted with NaOH [36].

Pipettes were filled with 3M KCl and had resistances of 0.3–3.0 MΩ. Experiments were performed with a Geneclamp 500B amplifier (Axon Instruments, Union City, CA, USA) and a Digidata 1322A interface (Axon Instruments, Union City, CA, USA) [37]. Data acquisition was achieved with pCLAMP 9.2 (Axon Instruments, Union City, CA, USA) [38]. The analysis of dual-electrode voltage clamp data was performed with Clampfit9.2 (Axon instruments, Union City, CA, USA) [39], [40]. The holding potential was kept at −80 mV and the outward tail currents were elicited by voltage pulses to a potential of −60 mV for 500 ms after preconditioning steps to potentials between −80 and +70 mV for 500 ms. Leak currents estimated from the tail current measured after the preconditioning prepulse to −80 mV were subtracted. For normalization, the individual tail currents at +70 mV were divided by the mean tail current at +70 mV of *Xenopus* oocytes expressing hERG alone.

To visualize hERG-HA protein abundance in *Xenopus* oocytes cell membrane, immunocytochemistry was performed in *Xenopus* oocytes injected with water and with hERG-HA with or without additional injection of wild-type B-RAF. After fixation in 4% paraformaldehyde/PBS for at least 4 hours, the oocytes were cryoprotected in 30% sucrose, frozen in mounting medium, and placed on a cryostat. Sections were collected at a thickness of 8 µm on coated slides and stored at −80°C. For immunostaining, slides were dried at room temperature, fixed in acetone/methanol (1:1) for 15 min, washed in PBS and blocked for 1 hour in 5% bovine serum albumin/PBS. The primary antibody used was rat monoclonal anti-HA antibody (1 µg/ml, clone 3 F10, Roche, Mannheim, Germany) incubated in a moist chamber overnight at 4°C. Binding of primary antibody was visualised with anti-rat Alexa488-conjugated antibody (diluted 1:200, Invitrogen, UK) for 1 hour at room temperature. The slides were mounted with ProLong Gold antifade reagent (Invitrogen, UK). Images were taken on a fluorescence laser scanning microscope (LSM 510; Carl Zeiss MicroImaging, Göttingen, Germany) with A-Plan 40×/1.2 W DICIII. Brightness and contrast settings were kept constant during imaging of all oocytes in each injection series. Due to autofluorescence of the oocyte yolk, unspecific immunofluorescence was observed inside the *Xenopus* oocytes.

For chemiluminescence experiments, *Xenopus* oocytes expressing hERG-HA with or without additional coexpression of wild-type B-RAF were blocked in 1% BSA/ND96 for 20 minutes at 4°C. Oocytes were incubated with 0.5 µg/ml primary rat monoclonal anti-HA antibody (clone 3 F10, Roche, Mannheim, Germany) and subsequently with secondary HRP-conjugated goat anti-rat antibody (diluted 1:1000, Cell Signaling Technology, MA, USA). Individual oocytes were placed in 96 well plates with 20 µl of SuperSignal ELISA Femto Maximum Sensitivity Substrate (Pierce, Rockford, IL, USA). The chemiluminescence of the single oocytes was quantified in a luminometer (Walter Wallac 2 plate reader, Perkin Elmer, Juegesheim, Germany) by integrating the signal over a period of 1 s. Results display normalized relative light units. Integrity of the measured oocytes was assessed by visual control after the measurement to avoid unspecific light signals from the cytosol.

Rhabdomyosarcoma RD cells (ATCC, LGC Standards GmbH, Wesel, Germany) were routinely cultured in Dulbecco's Modified Eagle Medium DMEM containing 4.5 g/l glucose (PAA Laboratories GmbH, Germany), supplemented with 10% fetal bovine serum (PAA Laboratories GmbH, Germany), 100 U/ml penicillin and 100 µg/ml streptomycin (PAA Laboratories GmbH, Germany). Where indicated cells were treated for 24 hours with 10 µM B-RAF inhibitor PLX-4720 (Selleck Chemicals, USA) dissolved in DMSO. Equal amounts of vehicle were used as control.

To analyze hERG cell membrane abundance, rhabdomyosarcoma RD cells were washed twice with ice-cold PBS and labelled with 250 µg/ml Sulfo-NHS-LC-biotin (Pierce, Rockford, IL, USA) in PBS for 30 minutes at 4°C. The Sulfo-NHS-LC-biotin bound to the membrane proteins was quenched with 50 mM Tris-HCl buffer pH 7.4. After washing, rhabdomyosarcoma RD cells were lysed with ice-cold RIPA buffer (Cell Signaling, Danvers, MA) supplemented with complete protease and phosphatase inhibitor cocktail (Thermo Fisher Scientific, Rockford, IL). After centrifugation at 10000 rpm for 5 min, 200 µg of proteins were supplemented with 50 µl washed immobilized Neutravidin Agarose beads (Pierce, Rockford, IL, USA) and incubated at 4°C overnight on a rotator. The beads were then pelleted by a 1 min centrifugation at 13000 rpm and washed 3 times in PBS containing 1% NP-40/0.1%SDS and twice in 0.1% NP-40/0.5 M NaCl. Proteins were solubilized in Roti-Load1 buffer (Carl Roth GmbH, Karlsruhe, Germany) at 95°C for 10 min, separated on 8% SDS-polyacrylamide gels and transferred to PVDF membranes. After blocking with 5% non-fat dry milk in TBS 0.1% Tween20 for 1 hour at RT, the blots were incubated overnight at 4°C with rabbit anti-K_v_11.1 (hERG, extracellular) antibody (diluted 1:200, Alamone Labs, Jerusalem, Israel). After washing (TBST), blots were incubated with anti-rabbit HRP-conjugated antibody (diluted 1:1000, Cell Signaling, Danvers, MA, USA) for 1 hour at RT. Antibody binding was detected with the ECL detection reagent (Amersham, Freiburg, Germany). Bands were quantified with Quantity One Software (Bio-Rad, Muenchen, Germany) and results are shown normalized to the control treated group.

For flow cytometry, rhabdomyosarcoma RD cells were washed once with PBS, detached from the plates by incubation for 10 minutes with Versene solution (Life Technologies, Gibco, USA) at 37°C, 5% CO_2_, and centrifuged for 5 minutes at 1200 rpm. 1×10^6^ cells in 25 µl of PBS were stained 20 min with 1 µl anti-Kv11.1 (hERG, extracellular)-FITC antibody (Alamone Labs, Jerusalem, Israel). The forward scatter of the cells was determined and hERG-FITC fluorescence intensity was measured in FL-1 with an excitation wavelength of 488 nm and an emission wavelength of 530 nm on a flow cytometer (FACSCalibur, BD Biosciences, USA). Results are shown as percentage of hERG-FITC positive cells normalized to the control treated group.

Patch clamp experiments were performed at RT in voltage clamp, fast-whole-cell mode according to Hamill et al. [41] as described earlier [42]–[45]. The currents were recorded by EPC-9 amplifier (Heka, Lambrecht, Germany) using Pulse software (Heka) and ITC-16 Interface (Instrutech, Port Washington, N.Y., USA). The currents were elicited by voltage pulses to a potential of −120 mV for 500 ms after preconditioning steps at potentials between −80 and +60 mV for 2 s. Leak currents estimated from the tail current measured after the preconditioning prepulse to −80 mV were subtracted. The currents were recorded with an acquisition frequency of 10 kHz and 3 kHz low-pass filtered. The liquid junction potential ΔE between pipette and bath solutions was estimated according to Barry and Lynch [46] and corrected. The cells were superfused with a bath solution containing (in mmol/l): 92 NaCl, 40 KCl, 2 CaCl_2_, 2 MgCl_2_, 5 glucose, 10 HEPES/NaOH, pH 7.4. The pipettes were filled with an internal solution at a [Ca^2+^]_i_ of 10^−7^ M (pCa 7) containing (in mM): 120 K-gluconate, 10 NaCl, 2 MgCl_2_, 4 CaCl_2_, 10 EGTA/KOH, 10 HEPES/KOH, 3 Mg-ATP, pH 7.3.

Data are provided as arithmetic means ± SEM, n represents the number of independent experiments or of *Xenopus* oocytes investigated. All oocyte experiments were repeated with at least three batches of oocytes. In all repetitions, qualitatively similar data were obtained. Results were tested for significance by using non-parametric Kruskal-Wallis test, unpaired Student's t-test or Mann-Whitney test, where appropriate. Only p<0.05 was considered statistically significant.

## Results

**Figure 1:**
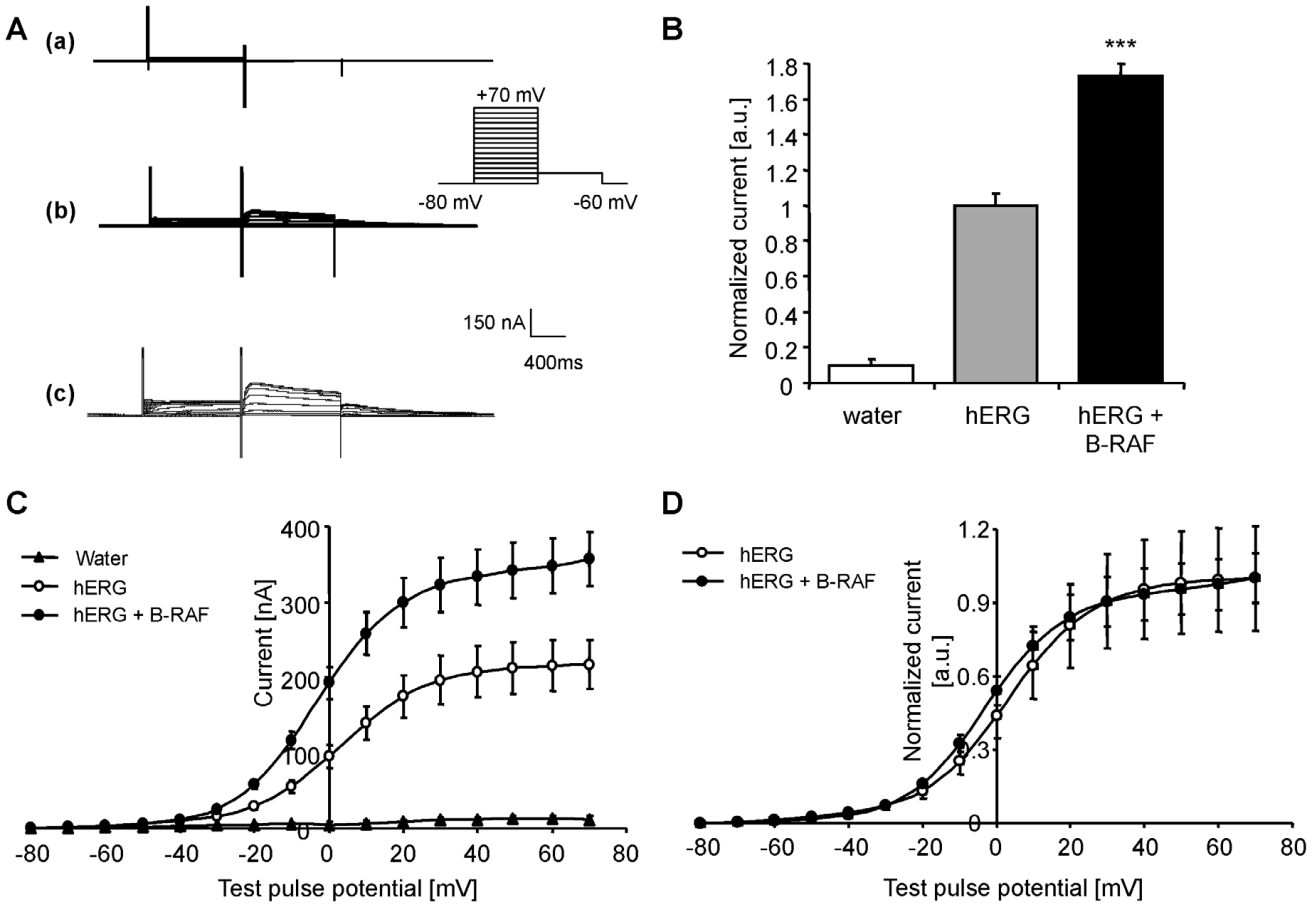
Coexpression of B-RAF increased hERG current in *Xenopus* oocytes. **A.** Original tracings recorded in *Xenopus* oocytes injected with water (a), with cRNA encoding hERG alone (b) or with cRNA encoding hERG together with wild-type B-RAF (c). The *Xenopus* oocytes were depolarized from −80 mV holding potential to different voltages followed by a 500 ms repolarization to −60 mV evoking outward tail currents. **B.** Arithmetic means ± SEM (n = 12–47, arbitrary units) of the normalized outward tail current following a depolarization to +70 mV, recorded in *Xenopus* oocytes injected with water (white bar), with cRNA encoding hERG alone (light grey bar), or with cRNA encoding both, hERG and wild-type B-RAF (black bar). ***(p<0.001) indicates statistically significant difference from *Xenopus* oocytes expressing hERG channels alone. **C.** Arithmetic means ± SEM (n = 12–47, nA) of the peak tail current as a function of voltage in *Xenopus* oocytes injected with water (black triangles), with cRNA encoding hERG alone (white circles) or with cRNA encoding hERG and wild-type B-RAF (black circles). **D.** Arithmetic means ± SEM (n = 22–47, arbitrary units) of the normalized peak tail current as a function of voltage in *Xenopus* oocytes injected with cRNA encoding hERG alone (white circles) or with cRNA encoding hERG together with wild-type B-RAF (black circles).

The present study explored whether the serine/threonine kinase B-RAF influences the activity of human ether-a-go-go related-gene K^+^ channels (hERG). In a first series of experiments, cRNA encoding hERG was injected either alone or together with cRNA encoding human wild-type B-RAF in *Xenopus* oocytes. As a control, the same amount of water was injected in *Xenopus* oocytes. HERG-mediated current was determined utilizing dual-electrode voltage clamp and the channel activity was analyzed by depolarization from −80 mV holding potential to different voltages followed by a 500 ms pulse to −60 mV. As illustrated in Fig. 1A and B, the tail current following injection of water in *Xenopus* oocytes was low in comparison with *Xenopus* oocytes injected with cRNA encoding hERG. Thus, *Xenopus* oocytes express low levels of channels displaying tail currents similar to those of hERG channels. Strong tail currents were observed, however, following injection of cRNA encoding hERG in *Xenopus* oocytes. HERG-mediated current was significantly enhanced by additional co-expression of wild-type B-RAF in hERG-expressing *Xenopus* oocytes (Fig. 1A and B). Fig. 1C illustrates the current-voltage relationship of hERG current with or without co-expression of wild-type B-RAF. The amplitude of the peak tail current was plotted as a function of the preceding test preconditioning potential. As illustrated in Fig. 1C, the absolute current values were up-regulated by co-expression of wild-type B-RAF. Following normalization to the maximum peak tail current for each group, no significant kinetic differences were apparent between *Xenopus* oocytes expressing hERG together with wild-type B-RAF and *Xenopus* oocytes expressing hERG alone (Fig. 1D). In other words, the voltage required for half maximal peak tail currents, as well as the activation threshold were similar in *Xenopus* oocytes expressing hERG alone and in *Xenopus* oocytes expressing hERG together with wild-type B-RAF.

**Figure 2:**
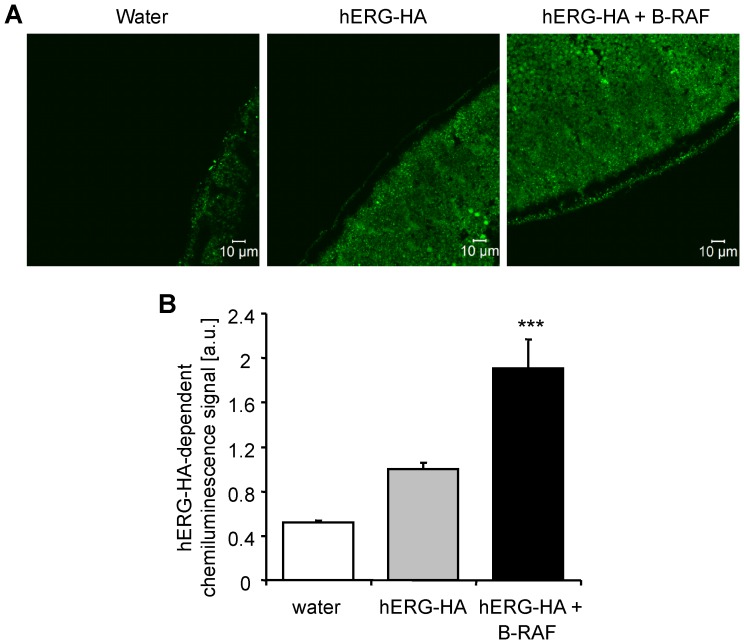
Coexpression of B-RAF increased hERG-HA protein abundance at the surface of hERG-expressing *Xenopus* oocytes. **A.** Confocal images of hERG-HA protein cell surface expression in *Xenopus* oocytes injected with water (left panel), with cRNA encoding hERG-HA alone (middle panel) or with cRNA encoding hERG-HA together with wild-type B-RAF (right panel). Images are representative of three independent experiments. **B.** Arithmetic means ± SEM (n = 81–93, arbitrary units) of hERG-HA protein abundance in the cell membrane measured by chemiluminescence in *Xenopus* oocytes injected with water (white bar), with cRNA encoding hERG-HA alone (light grey bar), or cRNA encoding hERG-HA and wild-type B-RAF (black bar). ***(p<0.001) indicates statistically significant difference from *Xenopus* oocytes expressing hERG channels alone.

At least in theory, B-RAF could up-regulate hERG activity by increasing hERG channel protein abundance in the *Xenopus* oocytes plasma membrane. In order to test that possibility, immunocytochemistry and confocal microscopy were applied to visualize hERG-HA protein in the cell membrane. As shown in Fig. 2A, the co-expression of hERG-HA with wild-type B-RAF was followed by an increase of hERG-HA protein abundance within the *Xenopus* oocyte cell membrane as compared to *Xenopus* oocytes expressing hERG-HA alone. In order to quantify hERG-HA protein abundance in the cell membrane of *Xenopus* oocytes, chemiluminescence was employed. As compared to *Xenopus* oocytes expressing hERG-HA alone, the co-expression of wild-type B-RAF was followed by a statistically significant increase of chemiluminescence reflecting hERG-HA protein abundance within the *Xenopus* oocyte cell membrane (Fig. 2B).

**Figure 3:**
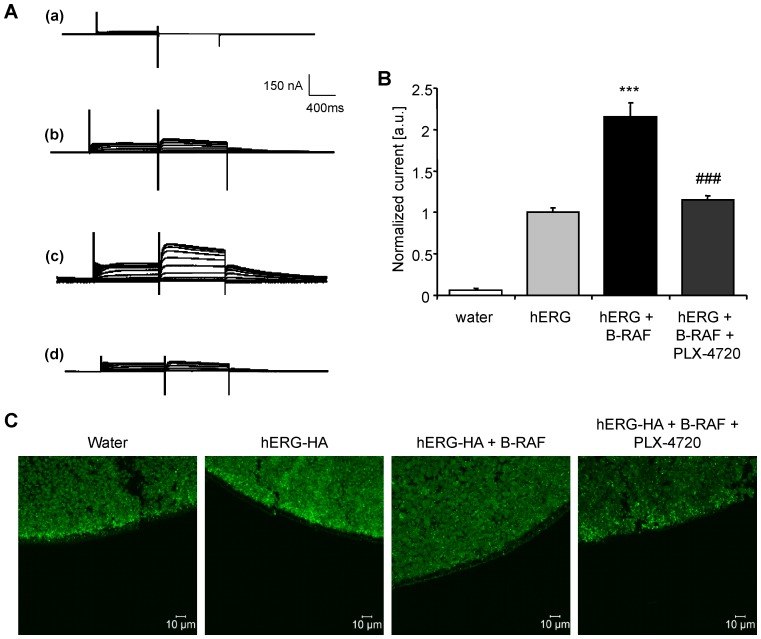
B-RAF inhibitor PLX-4720 decreased hERG current in *Xenopus* oocytes co-expressing hERG and B-RAF. **A.** Original tracings recorded in *Xenopus* oocytes injected with water (a), with cRNA encoding hERG alone (b) or with cRNA encoding hERG together with wild-type B-RAF without (c) and with (d) treatment with B-RAF inhibitor PLX-4720 (10 µM, 24 hours). The *Xenopus* oocytes were depolarized from −80 mV holding potential to different voltages followed by a 500 ms repolarization to −60 mV evoking outward tail currents. **B.** Arithmetic means ± SEM (n = 12–46, arbitrary units) of the normalized outward tail current following a depolarization to +70 mV, recorded in *Xenopus* oocytes injected with water (white bar), with cRNA encoding hERG alone (light grey bar), or with cRNA encoding hERG together with wild-type B-RAF without (black bar) and with (dark grey bar) treatment with B-RAF inhibitor PLX-4720 (10 µM, 24 hours). ***(p<0.001) indicates statistically significant difference from *Xenopus* oocytes expressing hERG channels alone; ###(p<0.001) indicates statistically significant difference from *Xenopus* oocytes expressing hERG together with B-RAF without treatment with PLX-4720. **C.** Confocal images of hERG-HA protein cell surface expression in *Xenopus* oocytes injected with water (first panel), with cRNA encoding hERG-HA alone (second panel) or with cRNA encoding hERG-HA together with wild-type B-RAF without (third panel) or with (last panel) treatment with B-RAF inhibitor PLX-4720 (10 µM, 24 hours). Images are representative of three independent experiments.

Further experiments elucidated the effect of the potent B-RAF inhibitor PLX-4720 on hERG-mediated current in B-RAF and hERG-expressing *Xenopus* oocytes. As illustrated in Fig. 3A and B, the hERG tail current in *Xenopus* oocytes expressing both, hERG and B-RAF was significantly decreased by treatment for 24 hours with 10 µM of the B-RAF inhibitor PLX-4720. No statistically significant difference was observed between hERG tail currents in *Xenopus* oocytes co-expressing hERG together with B-RAF and treated with 10 µM PLX-4720 and *Xenopus* oocytes expressing hERG alone. Along those lines treatment of hERG-HA and B-RAF-expressing *Xenopus* oocytes with 10 µM PLX-4720 was followed by a decline of hERG-HA cell surface protein abundance (Fig. 3C). Thus, PLX-4720 treatment fully reversed the effect of B-RAF on hERG cell surface protein expression and activity.

**Figure 4:**
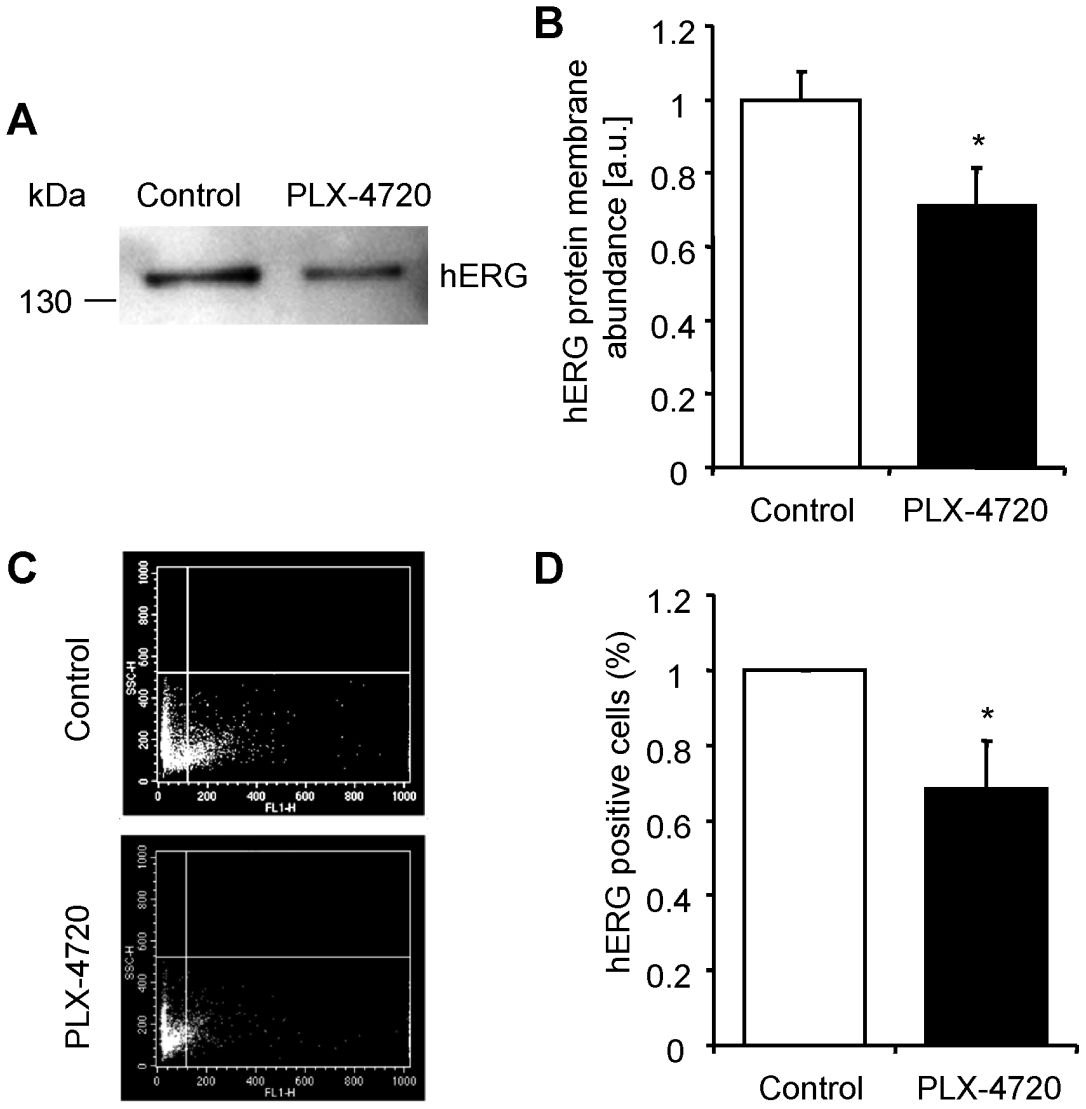
B-RAF inhibitor PLX-4720 decreased hERG protein abundance at the cell surface in rhabdomyosarcoma RD cells. **A.** Representative original western blot showing hERG membrane protein abundance (anti-K_v_11.1 antibody, Alamone Labs) analyzed by cell surface biotinylation in rhabdomyosarcoma RD cells after 24 hours treatment with vehicle alone (Control) or with 10 µM B-RAF inhibitor PLX-4720 (PLX-4720). **B.** Arithmetic means ± SEM (n = 7, arbitrary units) of normalized hERG membrane protein abundance analyzed by cell surface biotinylation in rhabdomyosarcoma RD cells after 24 hours treatment with vehicle alone (white bar) or with 10 µM B-RAF inhibitor PLX-4720 (black bar). *(p<0.05) indicates statistically significant difference from rhabdomyosarcoma RD cells treated with vehicle alone. **C.** Representative original dot plots of hERG-FITC positive cells at the cell surface analysed by flow cytometry in rhabdomyosarcoma RD cells after 24 hours treatment with vehicle alone (Control) or with 10 µM B-RAF inhibitor PLX-4720 (PLX-4720); FL-1 Height: hERG-FITC fluorescence intensity. **D.** Arithmetic means ± SEM (n = 5, %) of normalized percentage of positive cells showing hERG expression at the cell surface analyzed by flow cytometry in rhabdomyosarcoma RD cells after 24 hours treatment with vehicle alone (white bar) or with 10 µM B-RAF inhibitor PLX-4720 (black bar). *(p<0.05) indicates statistically significant difference from rhabdomyosarcoma RD cells treated with vehicle alone.

In another series of experiments we explored whether B-RAF similarly regulates the activity of the human ether-a-go-go related gene K^+^ channels (hERG) in rhabdomyosarcoma RD cells, which have previously been shown to express hERG channels [47], [48]. To this end, the rhabdomyosarcoma RD cells were treated for 24 hours with 10 µM of the B-RAF inhibitor PLX-4720 and hERG cell membrane protein abundance was analysed by biotinylation of the cell surface proteins with subsequent western blotting and by flow cytometry experiments. As illustrated in Fig. 4A and B, treatment of the rhabdomyosarcoma RD cells with B-RAF inhibitor PLX-4720 was followed by a statistically significant decrease in hERG cell membrane protein abundance as compared to rhabdomyosarcoma RD cells treated with vehicle alone. Moreover, as shown by flow cytometry measurements the number of hERG-FITC positive cells, i.e. rhabdomyosarcoma RD cells expressing hERG K^+^ channels at the cell surface, was significantly decreased following treatment with PLX-4720 (Fig. 4C and D). Thus, PLX-4720 treatment decreased hERG cell membrane protein abundance in rhabdomyosarcoma RD cells.

**Figure 5:**
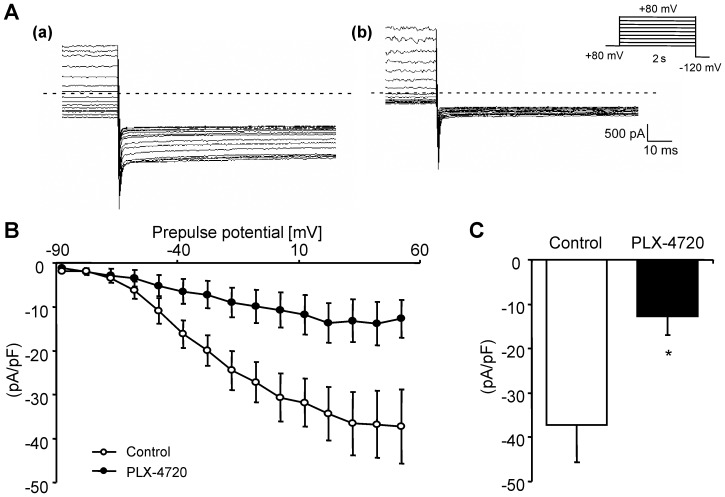
B-RAF inhibitor PLX4720 decreased hERG currents in rhabdomyosarcoma RD cells. **A.** Inward currents elicited in a bath solution containing 40: the membrane potential was held at −80 mV and then after the preconditioning step from −80 mV to +60 mV for 2 s stepped to the test potential of −120 mV for 500 ms. The currents were measured in rhabdomyosarcoma RD cells after 24 hours treatment with vehicle alone (a) or with 10 µM B-RAF inhibitor PLX-4720 (b). **B.** Mean peak current density ± SEM (n = 5–12) plotted against the precondition potential in rhabdomyosarcoma RD cells after 24 hours treatment with vehicle alone (white cycles) or with 10 µM B-RAF inhibitor PLX-4720 (black cycles). C. Mean peak current density ± SEM (n = 5–12) measured at −120 mV after the precondition potential to +50 mV in rhabdomyosarcoma RD cells after 24 hours treatment with vehicle alone (white bar) or with 10 µM B-RAF inhibitor PLX-4720 (black bar). *(p<0.05) indicates statistically significant difference from rhabdomyosarcoma RD cells treated with vehicle alone.

Similar observations were made with patch clamp experiments in rhabdomyosarcoma RD cells. As illustrated in Fig. 5, tail currents typical for hERG K^+^ channels were indeed observed in rhabdomyosarcoma RD cells. The hERG-mediated tail current was significantly lower in rhabdomyosarcoma RD cells treated for 24 hours with 10 µM PLX-4720 than in rhabdomyosarcoma RD cells treated with vehicle alone (Fig. 5C).

## Discussion

The present study reveals that the serine/threonine kinase B-RAF is a powerful stimulator of the human ether-a-go-go related-gene K^+^ channels (hERG). Co-expression of wild-type B-RAF increased hERG channel protein abundance in the cell membrane and thus increased the respective hERG-mediated current across the cell membrane in *Xenopus* oocytes. Furthermore, the down-regulation of hERG channel protein abundance and activity in rhabdomyosarcoma RD cells by treatment with the B-RAF inhibitor PLX-4720 points to a role of B-RAF in the regulation of hERG channels in those tumor cells. It must be kept in mind, though, that the selectivity of the inhibitor may be limited. The experiments in *Xenopus* oocytes demonstrate, however, that the inhibitor PLX-4720 reverses the effect of wild-type B-RAF co-expression and apparently does not influence hERG activity by mechanisms other than B-RAF inhibition.

The present study did not address the mechanisms involved in the regulation of hERG channel protein abundance. Even though our experiments do not rule out an effect of B-RAF on hERG transcription, the results in *Xenopus* oocytes indicate that wild-type B-RAF is at least partially effective through effects on channel insertion into the cell membrane and/or channel protein stability in the cell membrane. No putative consensus sequence specific for the B-RAF phosphorylation site recognition motif could be identified in the hERG protein sequence, suggesting that the effects may not depend on direct phosphorylation of the channels by B-RAF. Instead, B-RAF may be effective by influencing other regulators of hERG channels. Degradation of hERG channel protein is regulated by the ubiquitin ligase Nedd4-2 [48]–[51], which ubiquitinates target proteins thus preparing them for degradation [49], [51]. Moreover, at least in theory, B-RAF may be effective by influencing the activity of other kinases. Kinases involved in the effect of growth factors on hERG expression include protein tyrosine kinases [17], VEGFR-2 (KDR) kinase [19], serum- and glucocorticoid-inducible kinase isoforms SGK1 and SGK3 [27], [49], AMP-activated protein kinase [48] and phosphatidylinositol-3-phosphate-5-kinase PIKfyve [52]. Several additional factors and drugs acting on hERG channel trafficking to the cell membrane have been described including arsenic trioxide [53], [54], pentamidine [55], [56], probucol [57], the anti-arrhythmic drug E4031 [58], [59], cisapride or quinidine [58].

HERG channels are known regulators of tumor cell proliferation and apoptosis [9]–[11]. Thus, B-RAF-sensitive regulation of hERG channels may impact on proliferation, survival and migration of tumor cells [5], [6]. Regulation of channels by co-expressed signaling molecules in *Xenopus* oocytes may not necessarily reflect the effect of the respective signalling molecule on channel activity in tumor cells. The interaction of signalling molecules with channels may depend on the expression level of the channel and the signalling molecule, which may be different in cRNA injected *Xenopus* oocytes and defined mammalian cells. Moreover, additional signalling pathways expressed differently in *Xenopus* oocytes and mammalian cells may modify the interaction of the signalling molecule with the channels. The observed effect of the B-RAF inhibitor PLX-4720 strongly suggests, however, that B-RAF sensitivity of hERG channels is relevant in rhabdomyosarcoma RD cells. Thus, B-RAF-sensitive hERG K^+^ channel up-regulation possibly contributes to cell proliferation and apoptosis of tumor cells.

At least in theory, up-regulation of hERG K^+^ channels by B-RAF may further influence cardiac repolarization [1], [2]. Along those lines, cardiac cells express all the three RAF family members, RAF-1, B-RAF, and A-RAF, which are apparently important for the survival and growth of cardiomyocytes [60]. A negative influence of B-RAF inhibitors on cardiomyocytes function and survival may thus present an important potential side effect of B-RAF inhibitors [61].

In conclusion, the present study demonstrates that wild-type B-RAF is a powerful stimulator of the voltage-gated hERG K^+^ channels and may thus participate in the proliferation, survival and function of tumor cells and possibly cardiomyocytes.
